# Genomic Evaluation for a Crossbreeding System Implementing Breed-of-Origin for Targeted Markers

**DOI:** 10.3389/fgene.2019.00418

**Published:** 2019-05-03

**Authors:** Claudia A. Sevillano, Henk Bovenhuis, Mario P. L. Calus

**Affiliations:** ^1^Wageningen University & Research Animal Breeding and Genomics, Wageningen, Netherlands; ^2^Topigs Norsvin Research Center, Beuningen, Netherlands

**Keywords:** origin of alleles, crossbred, genomic prediction, finisher, pig

## Abstract

The genome in crossbred animals is a mosaic of genomic regions inherited from the different parental breeds. We previously showed that effects of haplotypes strongly associated with crossbred performance are different depending upon from which parental breed they are inherited, however, the majority of the genomic regions are not or only weakly associated with crossbred performance. Therefore, our objective was to develop a model that distinguishes between selected single nucleotide polymorphisms (SNP) strongly associated with crossbred performance and all remaining SNP. For the selected SNP, breed-specific allele effects were fitted whereas for the remaining SNP it was assumed that effects are the same across breeds (SEL-BOA model). We used data from three purebred populations; S, LR, and LW, and the corresponding crossbred population. We selected SNP that explained together either 5 or 10% of the total crossbred genetic variance for average daily gain in each breed of origin. The model was compared to a model where all SNP-alleles were allowed to have different effects for crossbred performance depending upon the breed of origin (BOA model) and to a model where all SNP-alleles had the same effect for crossbred performance across breeds (G model). Across the models, the heritability for crossbred performance was very similar with values of 0.29–0.30. With the SEL-BOA models, in general, the purebred-crossbred genetic correlation (r_pc_) for the selected SNP was larger than for the non-selected SNP. For breed LR, the r_pc_ for selected SNP and non-selected SNP estimated with the SEL-BOA 5% and SEL-BOA 10% were very different compared to the r_pc_ estimated with the G or BOA model. For breeds S and LW, there was not a big discrepancy for the r_pc_ estimated with the SEL-BOA models and with the G or BOA model. The BOA model calculates more accurate breeding values of purebred animals for crossbred performance than the G model when r_pc_ differs (≈10%) between the G and the BOA model. Superiority of the SEL-BOA model compared to the BOA model was only observed for SEL-BOA 10% and when r_pc_ for the selected and non-selected SNP differed both (≈20%) from the r_pc_ estimated by the G or BOA model.

## Introduction

The breeding goal of pig breeding programs is commonly to select purebred animals for improved performance of their crossbred descendants. It has been shown that using crossbred information, in addition to commonly used purebred information, improves the accuracy of selection. The benefit was observed using crossbred phenotypes either with pedigree (Wei and Van der Steen, 1991) and even more pronounced with crossbred genomic information (Xiang et al., 2017; Sewell et al., 2018). The most common genetic markers used for genomic selection are single nucleotide polymorphisms (SNP), i.e., bi-allelic markers. For crossbred animals, as their genome is a mosaic of genomic regions inherited from the different parental breeds, depending from which breed a SNP-allele was inherited, it might have different effects. These different allele effects can arise because: (1) quantitative trait loci (QTL) may be in linkage disequilibrium with different single nucleotide polymorphisms (SNP) depending from which parental breed the QTL was inherited (Lopes, 2016), (2) partly different quantitative trait nucleotides (QTN) could be underlying a QTL in different parental breeds, while the common QTN may have different allele frequencies in the different parental breeds, with the extreme case where it is not segregating in one or more breeds (Wientjes et al., 2015), and (3) epistatic interactions may differ between parental breeds (Mackay, 2014). In most previous studies using crossbred genomic information potential differences in SNP-allele effects due to the breed of origin were ignored (e.g., Hidalgo et al., 2015; Veroneze et al., 2015; Sewell et al., 2018). A model that accounts for breed of origin of alleles (BOA model), has been proposed by Dekkers (2007), Ibánez-Escriche et al. (2009), and Christensen et al. (2014). The BOA model was expected to be beneficial when using commercial crossbred genomic information for estimation of breeding values of purebred pigs for crossbred performance. The observed benefits of the BOA model, however, were limited to traits with low genetic correlation between purebred and crossbred performance (r_pc_) and to crossbred populations that originated from distantly-related breeds, as was shown in studies with simulated two-way (Ibánez-Escriche et al., 2009; Esfandyari et al., 2015) and three-way crossbred data (Ibánez-Escriche et al., 2009) and in studies with real two-way (Xiang et al., 2016) and three-way crossbred data (Sevillano et al., 2017).

The BOA model allows all SNP-alleles to have a different estimated effect for crossbred performance depending upon the breed of origin. In a recent study, Sevillano et al. (2018) confirmed that the effect of haplotypes strongly associated with crossbred performance are different depending upon from which population they originate. It was also shown, however, that the majority of the genomic regions are not or only weakly associated with crossbred performance. We hypothesized that targeting genomic regions strongly associated with crossbred performance and differentiating their SNP-allele effects according to their breed of origin, might improve prediction models for crossbred performance. Therefore, the objective of this study was to develop a model that accounts for breed-specific allele effects only for SNP strongly associated with crossbred performance, and for the rest of the SNP assumes that effects are the same across breeds. Thus, the model had one across-breed component, and a breed-specific component for each breed of origin. The performance of this model, in terms of estimated variances for the different model components and overall prediction accuracy, was tested using combined information from both purebred and three-way commercial crossbred pigs for average daily gain. The model was compared to the BOA model (allowing all SNP-alleles to have a different effect for crossbred performance depending upon the breed of origin) and a G model (all SNP-alleles having the same effect for crossbred performance across breeds).

## Materials and Methods

### Ethics Approval

The data used for this study was collected as part of routine data recording in a commercial breeding program. Samples collected for DNA extraction were only used for routine diagnostic purposes of the breeding program. Data recording and sample collection were conducted strictly in line with the Dutch law on the protection of animals (Gezondheids- en welzijnswet voor dieren).

### Data

The data consisted of three purebred pig populations; Synthetic boar (S), Landrace (LR), and Large White (LW), and one commercial crossbred population [S × (LR × LW) or S × (LW × LR)]. All pigs were genotyped using one of the three following SNP panels: Illumina PorcineSNP60.v2 BeadChip (60 K.v2), Illumina PorcineSNP60 BeadChip (60 K), or Illumina PorcineSNP10 BeadChip (10 K). Pigs genotyped with the 60 or 10 K chips were imputed to the 60 K.v2 panel using FImpute Version 2.2 software (Sargolzaei et al., 2014) with default parameter settings and using pedigree information. The imputation strategy was similar to Sevillano et al. (2016), where each of the three purebred populations, LR, LW, and S, were imputed in two steps: (1) pigs genotyped with the 10 K chip were imputed to 60 K, and (2) all pigs with 60 K data (imputed or genotyped) were imputed to 60 K.v2. For the commercial crossbred population, imputation was done in a single step, commercial crossbred pigs genotyped with the 10 K chip were directly imputed to 60 K.v2, because all ancestors were genotyped or already imputed to 60 K.v2.

Purebred pigs were located in nucleus farms while crossbred pigs were located in experimental farms representative of commercial production conditions. Phenotypes for average daily gain (ADG) were measured in purebred and commercial crossbred pigs. ADG for purebred pigs was calculated as the difference of on-test body weight at an average age of 60 days and off-test body weight at an average age of 173 days divided by the number of days. ADG for commercial crossbred pigs was calculated as the difference of on-test body weight at an average age of 70 days of age and body weight at the end of the finishing period, which was on average 120 kg, divided by the number of days.

The numbers of available genotypes and phenotypes were 7,575, 3,288 and 12,794 for purebred population S, LR, and LW, respectively, and 2,816 for the commercial crossbred population. For all pigs, four generations of pedigree information were included for analysis.

### Proposed Model

The proposed model considers breed-specific effects only for SNP strongly associated with crossbred performance, and for the remaining SNP assumes that effects are the same across breeds. To build this model, we first needed to determine the breed of origin of alleles in crossbred pigs and secondly, determine which SNP are strongly associated with crossbred ADG. In this section, we will firstly introduce the proposed model, followed by a subsection “Inference of the breed of origin of alleles” where we explain how we determined the breed of origin of alleles in crossbred pigs, and we finish with a subsection “Targeting SNP” where we explain how we determine which are the SNP strongly associated with ADG performance in crossbred pigs. Hereafter, we will refer to the SNP strongly associated with crossbred performance as “selected SNP” and to the remaining SNP as “non-selected SNP.”

#### The Model

To model breed-specific effects for SNP strongly associated with crossbred performance and across-breed effects for all other SNP, the following four-trait animal model was fitted (SEL-BOA model):

$$\begin{array}{l} \;y_{S}=X_{S}b_{S}+W_{S}u_{S}+Z_{S}a_{S}^{sel}+Z_{S}a_{S}^{non-sel}+\;e_{S},\\ y_{LR}=X_{LR}b_{LR}+W_{LR}u_{LR}+Z_{LR}a_{LR}^{sel}+Z_{LR}a_{LR}^{non-sel}+\;e_{LR},\\ y_{LW}=X_{LW}b_{LW}+W_{LW}u_{LW}+Z_{LW}a_{LW}^{sel}+Z_{LW}a_{LW}^{non-sel}+\;e_{LW},\\ y_{CB}\;=X_{CB}b_{CB}+W_{CB}u_{CB}+Z_{CB}g_{CB\left(s\right)}^{sel}\\ \;+Z_{CB}g_{CB(LR)}^{sel}+Z_{CB}g_{CB(LW)}^{sel}+Z_{CB}a_{CB}^{non-sel}+\;e_{CB}, \end{array}$$

where **y**_**S**_, **y**_**LR**_, **y**_**LW**_, and **y**_**CB**_ are the vectors of the phenotypes for S, LR, LW, and commercial crossbred pigs, respectively; **b**_**S**_, **b**_**LR**_, **b**_**LW**_, **b**_**CB**_ represent the vectors of fixed effects for farm^*^breed^*^sex and birth weight as covariable and **X**_**S**_, **X**_**LR**_, **X**_**LW**_, **X**_**CB**_ are the respective incidence matrices relating pig phenotypes to fixed effects; **u**_**S**_, **u**_**LR**_, **u**_**LW**_, **u**_**CB**_ represent the vectors of random common litter effects, and **W**_**S**_, **W**_**LR**_, **W**_**LW**_, **W**_**CB**_ are the respective incidence matrices relating pig phenotypes to litter effects; ${\text{a}}_{\text{S}}^{\text{s}e\text{l}}$, ${\text{a}}_{\text{L}\text{R}}^{\text{s}e\text{l}}$, ${\text{a}}_{\text{L}\text{W}}^{\text{s}e\text{l}}$, are the vectors of additive genetic effects in purebred pigs due to the selected SNP, ${\text{g}}_{\text{C}\text{B}(\text{S})}^{\text{s}e\text{l}}$, ${\text{g}}_{\text{C}\text{B}(\text{L}\text{R})}^{\text{s}e\text{l}}$, ${\text{g}}_{\text{C}\text{B}(\text{L}\text{W})}^{\text{s}e\text{l}}$ are the vectors of the additive genetic effect of purebred gametes in commercial crossbreds due to the selected SNP, ${\text{a}}_{\text{S}}^{\text{n}on-se\text{l}}$, ${\text{a}}_{\text{L}\text{R}}^{\text{n}on-se\text{l}}$, ${\text{a}}_{\text{L}\text{W}}^{\text{n}on-se\text{l}}$, are the vectors of additive genetic effects in purebred pigs considering only the non-selected SNP, ${\text{a}}_{\text{C}\text{B}}^{\text{n}on-se\text{l}}$ is the vector of additive genetic effect in commercials crossbred considering only the non-selected SNP, and **Z**_**S**_, **Z**_**LR**_, **Z**_**LW**_, **Z**_**CB**_ are the respective incidence matrices. Finally, **e**_**S**_, **e**_**LR**_, **e**_**LW**_, **e**_**CB**_ represent the vectors of random residual effects. The variance-covariance of the common litter effect was:

$$\begin{array}{l} \text{Var}\left[\begin{array}{c} {\text{u}}_{\text{S}}\\ \begin{array}{c} {\text{u}}_{\text{L}\text{R}}\\ {\text{u}}_{\text{L}\text{W}} \end{array}\\ {\text{u}}_{\text{C}\text{B}} \end{array}\right]=\left[\begin{array}{cccc} {{\text{I}}_{u_{S}}\sigma }_{u_{S}}^{2} & 0 & 0 & 0\\ 0 & {{\text{I}}_{u_{LR}}\sigma }_{u_{LR}}^{2} & 0 & 0\\ 0 & 0 & {{\text{I}}_{u_{LW}}\sigma }_{u_{LW}}^{2} & 0\\ 0 & 0 & 0 & {\text{I}}_{u_{CB}}\sigma_{u_{CB}}^{2}\\ \; \end{array}\right], \end{array}$$

and for the residual effect was:

$$\begin{array}{l} \text{Var}\left[\begin{array}{c} {\text{e}}_{\text{S}}\\ \begin{array}{c} {\text{e}}_{\text{L}\text{R}}\\ {\text{e}}_{\text{L}\text{W}} \end{array}\\ {\text{e}}_{\text{C}\text{B}} \end{array}\right]=\left[\begin{array}{cccc} {{\text{I}}_{e_{S}}\sigma }_{e_{S}}^{2} & 0 & 0 & 0\\ 0 & {\text{I}}_{e_{LR}}\sigma_{e_{LR}}^{2} & 0 & 0\\ 0 & 0 & {{\text{I}}_{e_{LW}}\sigma }_{e_{LW}}^{2} & 0\\ 0 & 0 & 0 & {\text{I}}_{e_{CB}}\sigma_{e_{CB}}^{2}\\ \; \end{array}\right]. \end{array}$$

The variance-covariance of additive genetic effect for breed S origin based on selected SNP was:

$$\begin{array}{lll} \text{Var}\left[\begin{array}{c} {\text{a}}_{\text{S}}^{\text{s}e\text{l}}\\ \begin{array}{c} {\text{a}}_{\text{C}\text{B}(\text{S})}^{\text{s}e\text{l}}\\ {\text{g}}_{\text{S}}^{\text{s}e\text{l}} \end{array}\\ {\text{g}}_{\text{C}\text{B}(\text{S})}^{\text{s}e\text{l}} \end{array}\right] & = & \left[\begin{array}{cc} {\text{σ}}_{{\text{a}}_{\text{S}}^{\text{sel}}}^{2} & \sigma_{{\text{a}}_{\text{S}}^{\text{sel}},{\text{g}}_{\text{S}}^{\text{sel}}}\\ {\text{σ}}_{{\text{a}}_{\text{S}}^{\text{sel}},{\text{g}}_{\text{S}}^{\text{sel}}} & {\text{σ}}_{{\text{g}}_{\text{S}}^{\text{sel}}}^{2} \end{array}\right]⊗{\text{G}}_{(\text{S})}^{\text{sel}}\\ = & \left[\begin{array}{cc} \sigma_{{\text{a}}_{\text{S}}^{\text{sel}}}^{2} & {\text{σ}}_{{\text{a}}_{\text{S}}^{\text{sel}},{\text{g}}_{\text{S}}^{\text{sel}}}\\ {\text{σ}}_{{\text{a}}_{\text{S}}^{\text{sel}},{\text{g}}_{\text{S}}^{\text{sel}}} & {\text{σ}}_{{\text{g}}_{\text{S}}^{\text{sel}}}^{2} \end{array}\right]⊗\left[\begin{array}{cc} {\text{G}}_{\text{S},\text{S}}^{\text{sel}} & {\text{G}}_{\text{S},\text{CB}(\text{S})}^{\text{sel}}\\ {\text{G}}_{\text{CB}(\text{S}),\text{S}}^{\text{sel}} & {\text{G}}_{\text{CB}\left(\text{S}\right),\text{CB}(\text{S})}^{\text{sel}}\\ \; \end{array}\right], \end{array}$$

where purebred S pigs have additive effects based on selected SNP, ${\text{a}}_{\text{S}}^{\text{s}e\text{l}}$ for purebred performance, and ${\text{a}}_{\text{C}\text{B}(\text{S})}^{\text{s}e\text{l}}$ for crossbred performance. The commercial crossbred pigs have additive effects based on selected SNP and based on gametes coming from breed S, ${\text{g}}_{\text{C}\text{B}(\text{S})}^{\text{s}e\text{l}}$ for crossbred performance, and ${\text{g}}_{\text{S}}^{\text{s}e\text{l}}$ for purebred performance. This last effect, ${\text{g}}_{\text{S}}^{\text{s}e\text{l}}$, is an artificial random vector that is added to be able to define the variance-covariance of additive genetic effects with the above Kronecker product, but does not have practical relevance (Christensen et al., 2015). The matrix ${\text{G}}_{(\text{S})}^{\text{s}e\text{l}}$ is a breed-specific partial relationship matrix for breed S which contains four blocks, one within S pigs (${\text{G}}_{\text{S},\text{S}}^{\text{s}e\text{l}}$), two between S and commercial crossbred pigs (${\text{G}}_{\text{S},\text{C}\text{B}(\text{S})}^{\text{s}e\text{l}}$and ${\text{G}}_{\text{C}\text{B}\left(\text{S}\right),\text{S}}^{\text{s}e\text{l}}$), and one within commercial crossbred pigs (${\text{G}}_{\text{C}\text{B}\left(\text{S}\;\right),\text{C}\text{B}(\text{S})}^{\text{s}e\text{l}}$).

The variance-covariance structures for breeds LR and LW are defined similarly. Therefore, the total additive genetic effect, based on selected SNP, in commercial crossbred pigs for crossbred performance is made up of ${\text{g}}_{\text{C}\text{B}(\text{S})}^{\text{s}e\text{l}}$, ${\text{g}}_{\text{C}\text{B}(\text{L}\text{R})}^{\text{s}e\text{l}}$, and ${\text{g}}_{\text{C}\text{B}(\text{L}\text{W})}^{\text{s}e\text{l}}$. There are six selected SNP genetic variance components, one for purebred and one for crossbred performance for each breed of origin, and three covariance components, one for each breed of origin. The three variance-covariance structures are assumed independent, i.e., no covariances are considered between S, LR, and LW effects (Christensen et al., 2015). The degree of allelic differentiation estimated with Weir and Cockerham's F_ST_ (Weir and Cockerham, 1984), was previously estimated among the three purebred populations by Sevillano et al. (2017) and were equal to 0.17 between S and LR, 0.12 between S and LW, and 0.14 between LW and LR, which indicates that they are distantly-related breeds, therefore it seems appropriate to assume no relationships between these purebred populations. Moreover, results from Xiang et al. (2017) using a **H**^**−1**^ relationship matrix with metafounders, and Sevillano et al. (2017) using a **G** relationship matrix, demonstrated that considering genomic relationships and covariances between purebred lines hardly affects the results of models for predicting crossbred performance.

To construct the three breed-specific partial relationship matrices, ${\text{G}}_{(\text{S})}^{\text{s}e\text{l}}$, ${\text{G}}_{(\text{L}\text{R})}^{\text{s}e\text{l}}$, and ${\text{G}}_{(\text{L}\text{W})}^{\text{s}e\text{l}}$, we used the breed of origin of phased alleles in commercial crossbred pigs. Then, the breed-specific partial relationship submatrices are defined as, e.g., breed S origin:

$$\begin{array}{l} \;G_{S,S}^{sel}=\left(M_{S}^{sel}-21p_{S^{\prime }}\right){\left(M_{S}^{sel}-21{p^{\prime }}_{S}\right)}^{\prime }{\left({\text{F}}_{\text{S}}\right)}^{-1},\\ \;G_{S,CB(S)}^{sel}=\left(M_{S}^{sel}-21{p^{\prime }}_{S}\right){\left(M_{CB(S)}^{sel}-1p^{S}{}^{\prime }\right)}^{\prime }{\left({\text{F}}_{\text{S}}\right)}^{-1},\text{and}\\ G_{CB\left(S\right),CB(S)}^{sel}=\left(M_{CB(S)}^{sel}-1{p^{\prime }}_{S}\right){\left(M_{CB(S)}^{sel}-1{p^{\prime }}_{S}\right)}^{\prime }{\left({\text{F}}_{\text{S}}\right)}^{-\;1} \end{array}$$

where ${\text{M}}_{\text{S}}^{\text{s}e\text{l}}\;$is a matrix containing breed-specific allele content of selected SNP for purebred S pigs (coded as 0, 1, or 2). ${\text{M}}_{\text{C}\text{B}(\text{S})}^{\text{s}e\text{l}}$ is a matrix containing breed S allele content of selected SNP for commercial crossbred pigs (coded as 0, or 1), so that alleles not assigned to breed S as breed of origin were set to missing, meaning that they had an entry of zero in the centered matrix represented by $\left({\text{M}}_{\text{C}\text{B}(\text{S})}^{\text{s}e\text{l}}-{1\text{p}}_{\text{S}}^{\prime }\right)$ and therefore effectively did not contribute to the computed breed S partial relationship; **p**_**S**_ is the vector of breed S specific frequencies of the counted allele ($p_{j}^{s})$, where $p_{j}^{s}$ was calculated across S and commercial crossbred pigs by counting the occurrences of alleles originating from the S breed and coded as 1, divided by the total number of S alleles in the S and commercial crossbred pigs on locus *j*. Finally, the scaling factor was defined as $\text{F\_S}=\underset{j}{\sum }2p_{j}^{S}\left(1-p_{j}^{S}\right)$, such that diagonal elements of an individual reflected its breed proportion for e.g., the S line, with expected values of 0.5 for the crossbreds and 1.0 for the purebred animals. The breed-specific partial relationship submatrices ${\text{G}}_{(\text{L}\text{R})}^{\text{s}e\text{l}}$ and ${\text{G}}_{(\text{L}\text{W})}^{\text{s}e\text{l}}$ are defined similarly to ${\text{G}}_{(\text{S})}^{\text{s}e\text{l}}$. However, the entries of the ${\text{M}}_{\text{C}\text{B}(\text{L}\text{R})}^{\text{s}e\text{l}}$ matrix containing the breed LR allele content for commercial crossbred pigs are set to a missing value if the origin of the allele corresponds to the other maternal line, and effectively does not contribute to the breed-specific partial relationship matrix for LR. The same applies for the ${\text{M}}_{\text{C}\text{B}(\text{L}\text{W})}^{\text{s}e\text{l}}$ matrix.

For additive genetic effects in commercial crossbred pigs based on non-selected SNP we did not model breed-specific allele effects and therefore this was defined by one vector, ${\text{a}}_{\text{C}\text{B}}^{\text{n}on-se\text{l}}$. The variance-covariance matrix of genetic effects based on non-selected SNP was:

$$\begin{array}{l} \;G_{S,S}^{sel}=\left(M_{S}^{sel}-21{p^{\prime }}_{S}\right){\left(M_{S}^{sel}-21{p^{\prime }}_{S}\right)}^{\prime }{\left({\text{F}}_{\text{S}}\right)}^{-1},\\ \;G_{S,CB(S)}^{sel}=\left(M_{S}^{sel}-21{p^{\prime }}_{S}\right){\left(M_{CB(S)}^{sel}-1p^{S^{\prime }}\right)}^{\prime }{\left({\text{F}}_{\text{S}}\right)}^{-1},\text{and}\\ G_{CB\left(S\right),CB(S)}^{sel}=\left(M_{CB(S)}^{sel}-1{p^{\prime }}_{S}\right){\left(M_{CB(S)}^{sel}-1{p^{\prime }}_{S}\right)}^{\prime }{\left({\text{F}}_{\text{S}}\right)}^{-\;1}, \end{array}$$

The genomic relationship matrix (**G**^***non*−*sel***^) was constructed using the first method in VanRaden (2008):

$$\text{Var}\left[\begin{array}{l} a_{S}^{non-sel}\\ a_{LR}^{non-sel}\\ a_{LW}^{non-sel}\\ a_{CB}^{non-sel} \end{array}\right]=\left[\begin{array}{llll} \;\sigma_{{\text{a}}_{\text{S}}}^{2} & \sigma_{{\text{a}}_{\text{S}},a_{\text{LR}}} & \sigma_{{\text{a}}_{\text{S}},{\text{a}}_{\text{LW}}} & \sigma_{{\text{a}}_{\text{S}},{\text{a}}_{\text{CB}}}\\ \sigma_{{\text{a}}_{\text{S}},{\text{a}}_{\text{LR}}} & \;\sigma_{{\text{a}}_{\text{LR}}}^{2} & \sigma_{{\text{a}}_{\text{LR}},{\text{a}}_{\text{LW}}} & \sigma_{{\text{a}}_{\text{LR}},{\text{a}}_{\text{CB}}}\\ \sigma_{{\text{a}}_{\text{S}},{\text{a}}_{\text{LW}}} & \sigma_{{\text{a}}_{\text{LR}},{\text{a}}_{\text{LW}}} & \;\sigma_{{\text{a}}_{\text{LW}}}^{2} & \sigma_{{\text{a}}_{\text{LW}},{\text{a}}_{\text{CB}}}\\ \sigma_{{\text{a}}_{\text{S}},{\text{a}}_{\text{CB}}} & \sigma_{{\text{a}}_{\text{LR}},{\text{a}}_{\text{CB}}} & \sigma_{{\text{a}}_{\text{LW}},{\text{a}}_{\text{CB}}} & \;\sigma_{{\text{a}}_{\text{CB}}}^{2} \end{array}\right]⊗G^{non-sel}.$$

where **M**^***non*−*sel***^ is a matrix containing non-selected SNP genotypes for each pig (coded as 0, 1, or 2), **p** is the vector of the frequencies of the counted allele (*p*_*j*_**)** calculated across the entire genotyped population, and the scaling factor was defined as $\text{F}=\underset{j}{\sum }2p_{j}\left(1-p_{j}\right)$.

The SEL-BOA model was implemented in the MiXBLUP software (Ten Napel et al., 2016). To estimate the variance components we used the same SEL-BOA model in the MTG2 software (Lee and Van der Werf, 2016).

#### Inference of the Breed of Origin of Alleles

To infer the breed of origin of alleles in crossbred pigs we used the BOA approach developed by Vandenplas et al. (2016) using the parameter settings recommended by Sevillano et al. (2016). The BOA approach consists of three steps: (1) Phasing the haplotypes of both purebred and commercial crossbred pigs with AlphaPhase1.1 software (Hickey et al., 2011). Phasing was performed using pedigree because it was available, however, phasing with AlphaPhase 1.1 software can be performed without using pedigree while obtaining similar results but demanding more computation time (Sevillano et al., 2016). Phasing was performed 18 times using nine different combinations of haplotype length and each combination was run both considering “Offset” and “NotOffset” modes, the “Offset” mode shifts the start of the cores to halfway along the first core, creating 50% overlap between cores. These settings allowed each allele to be considered 18 times through different haplotypes of variable length. (2) Determining the unique haplotypes among the purebred pigs. For assigning a breed of origin to a haplotype, at least 80% of its copies were required to be observed in a specific breed. (3) Assigning the breed of origin for each allele carried on the haplotypes of commercial crossbred pigs based on the knowledge of the breed of origin of the haplotypes, on the zygosity (i.e., homozygosity or heterozygosity) of the locus, and on the breed composition of the crossbred. Alleles that were not assigned a breed of origin were set to missing. SNP for which the paternal or maternal allele was assigned a breed of origin in < 90% of the cases were removed. Commercial crossbred pigs with assigned breed of origin for < 90% of their genome were removed. If an allele was observed < 5 times in one of the three breed of origin in the purebred populations or in the commercial crossbred population, the corresponding SNP was also removed from the final set of SNP. The final SNP set for subsequent analyses consisted of 41,529 SNP. All populations were analyzed with the same set of SNP.

#### Targeting SNP

Estimates for breed-specific SNP allele substitution effects were obtained from Sevillano et al. (2018) where they used a genomic BLUP with breed-specific partial relationship matrices (BOA model) (Sevillano et al., 2017). With this approach, genomic estimated breeding values (GEBV) for crossbred performance were calculated, and afterwards converted to SNP-allele effects by breed of origin. The BOA model allows all SNP to have breed-specific alleles. Therefore, it is similar to the SEL-BOA, however, for each breed the BOA-model only has the breed-specific component. GEBV of purebred pigs for crossbred performance (${\hat{\text{a}}}_{\text{C}\text{B}}$) were then converted to SNP-allele effects (${\hat{\alpha }}_{\text{C}\text{B}(\text{S})}$), e.g., for breed S using:

$$\begin{array}{l} {\hat{\text{a}}}_{\text{C}\text{B}\left(\text{S}\right)}=\;{\text{V}}_{\text{S}}{\hat{\alpha }}_{\text{C}\text{B}\left(\text{S}\right)}, \end{array}$$

where **V**_**S**_ contains centered genotypes for purebred S pigs and ${\hat{\alpha }}_{\text{C}\text{B}(\text{S})}$ are allele substitution effects, which can be obtained, respectively, by:

$$\begin{array}{l} \;V_{S}\;=\left(M_{S}\;-21p^{S}{}^{\prime }\right)\text{and}\\ {\hat{\alpha }}_{CB\left(\text{S}\right)}=V_{S}{}^{\prime }{\left(V_{S}{V^{\prime }}_{S}\right)}^{-1}{\hat{a}}_{CB\left(\text{S}\right)}={\left(F^{S}\right)}^{-1}{V^{\prime }}_{S}{\left(G_{S,S}\right)}^{-\;1}{\hat{a}}_{CB\left(\text{S}\right)} \end{array}$$

SNP-allele effects for crossbred performance of the other purebred populations were calculated similarly.

Afterwards, Sevillano et al. (2018) calculated the proportion of variance explained by a group of SNP in non-random association, called LD blocks [see Sevillano et al. (2018) for details on how LD blocks were built]. In a GBLUP model, all SNP are considered simultaneously in the model, therefore, the effect of a QTL is likely distributed across all SNP that have a non-random association with the QTL. For this reason, it is recommended to calculate the proportion of variance explained by a group of SNP in non-random association instead of reporting effects of single SNP (Lopes, 2016). LD blocks were built per breed of origin, therefore, non-random association between alleles at two loci was tested in the commercial crossbred population between all pair of loci coming from the same breed of origin. Percentage of genetic variance for crossbred performance explained by the *i*-th LD block was calculated as in Wang et al. (2014):

$$\frac{Var\left(a_{i}\right)}{\sigma_{a}^{2}}\times \frac{x_{n}}{n}\times 100\%=\frac{Var(\sum_{j=1}^{n}z_{j}{\hat{\alpha }}_{j})}{\sigma_{a}^{2}}\times \frac{x_{n}}{n}\times \;100\%,$$

where *a*_*i*_ is the genetic value of the *i*-th LD block, $\sigma_{a}^{2}$ is the total genetic variance for crossbred performance, **z**_***j***_ is a vector of gene content of the *j*-th SNP for all purebred individuals of the same breed, ${\hat{\alpha }}_{j}$ is the estimated effect for crossbred performance of the *j*-th SNP within the *i*-th LD block that contains *n* SNP, and x_n_ is the mean number of SNP across LD blocks. The factor $\frac{\text{x\_n}}{\text{n}}$ adjusts explained variances for the number of SNP included in the LD block.

For selecting SNP to be considered to have breed-specific allele effects, we took the top LD blocks that explained together at the most either 5 or 10% of the total additive genetic variance for crossbred performance in each breed of origin. Selected LD blocks per breed of origin were merged in one group and all the SNP in each of the selected LD blocks were then classified as selected SNP so their effects would be estimated in the SEL-BOA model as breed-specific. The non-selected SNP were assumed to have the same effect across the three breeds of origin, as outlined before. The SEL-BOA model was then ran two times, considering 5 and 10% of all SNP as selected SNP (SEL-BOA 5% and SEL-BOA 10% models).

### Cross-Validation

#### Comparison of Models

For comparison to the SEL-BOA model, we also calculated GEBV of purebred pigs for crossbred performance using two other four-trait animal models: the BOA model and the G model. The BOA model allowed all SNP-alleles to have a different effect for crossbred performance depending upon the breed of origin; to achieve this, the vectors of the additive genetic effect of purebred gametes in commercial crossbreds, i.e., **g**_**CB**(**S**)_, **g**_**CB**(**LR**)_, and **g**_**CB**(**LW**)_, considered all SNP. The G model considered all SNP-alleles having the same effect for crossbred performance across breeds, such that only one set of additive genetic effect in commercials crossbred was estimated (e.g., vector **a**_**CB**_).

#### Training Set

The accuracy of GEBV of purebred pigs for crossbred performance from all models was evaluated as the average accuracy obtained from 4-fold cross-validation. Because of different degrees of relationship between purebreds and commercial crossbred pigs, each of the four populations were first divided into four mutually exclusive clusters, using the K-means clustering method applied to a dissimilarity matrix computed from elements of the **G** matrix (Saatchi et al., 2011). The commercial crossbred pigs were not evenly distributed across the four clusters, therefore the clusters were reorganized to contain each more or less $\frac{1}{4}$ of the commercial crossbred pigs with the closest relationship (i.e., highest average relationship) based on the **G** matrix. Then, within each breed, each of the four crossbred clusters was assigned to one of the four purebred clusters with the closest relationship (i.e., highest average relationship) based on the **G** matrix to form a fold. Therefore, each fold contains one purebred cluster and one crossbred cluster. This way, for each breed, we obtained 4-folds to be included in the cross-validation.

In each training analysis, the data excluded phenotypes of purebred and commercial crossbred pigs from 1-fold to train on the remaining 3-folds to predict GEBV for crossbred performance of the excluded purebred pigs (validation set). This resulted in every purebred pig having GEBV for crossbred performance that were obtained without using performance of the most closely-related commercial crossbred pigs for training. Thus, the information coming from the most closely-related commercial crossbred pigs could be used for validation. The number of pigs in the validation and training sets for each of the folds of the cross-validation are in Table 1.

**Table 1:** Cross-validation strategy for performance of average daily gain in crossbreds.

**Fold**	**Training**				**Validation**			
	**S**	**LR**	**LW**	**CB**	**S**	**LR**	**LW**	**CB**
1	5,365	2,624	9,061	2,112	2,183	665	3,738	704
2	5,771	2,329	8,194	2,117	1,777	960	4,605	699
3	6,017	2,188	10,327	2,109	1,531	1,101	2,471	707
4	5,491	2,726	10,815	2,110	2,057	562	1,980	706

*Numbers of individuals for Synthetic boar (S), Landrace (LR), Large White (LW), and three-way crossbred (CB) pigs*.

#### Validation Set

For the purebred pigs used for the validation, some sort of phenotype is needed to be able to compute the prediction accuracy. Purebred pigs cannot have an own performance for crossbred performance. In our data they did not have large offspring groups, needed to compute average offspring performance as an accurate phenotype. Therefore, we calculated deregressed proofs (DRP) for purebred pigs within the validation sets to validate the predictions of our models. For this, first we obtained estimated breeding values (EBV) from the four-trait model with a pedigree-based relationship matrix. This resulted in an EBV for crossbred performance for each purebred pig. The EBV were estimated based on performance of the commercial crossbred pigs assigned to each of the validation folds (Table 1). Within each validation fold, the EBV of purebred pigs for crossbred performance were then deregressed according to Calus et al. (2016). The deregression involved removal of all effects of relatives in the same validation set, and correction for regression to the mean, to obtain a more accurate estimate of the expected phenotype. In addition, a weighting factor (*w*) was estimated for each DRP value based on the reliability of the calculated DRP. These *w* are the effective record contributions (Pribyl et al., 2013), and reflect the amount of information in the DRP contributed by the animal's crossbred relatives, correcting for any information of the crossbred relatives of other purebred animals that contributed to its EBV before deregression.

#### Predictive Ability

Accuracies of all models were calculated as the weighted correlation between the DRP and the GEBV of purebred pigs for crossbred performance, where the weighting factor *w* was used to account for differences in the amount of available information on relatives to estimate DRP. The standard error (SE) of the correlations were approximated as $\frac{1-{\text{r}}^{2}}{\sqrt{\text{N}}}$, were *r* is the estimated correlation of the model, and N is the number of validation animals (Stuart and Ord, 1994).

## Results

### Targeted SNP

**Figure 1:**
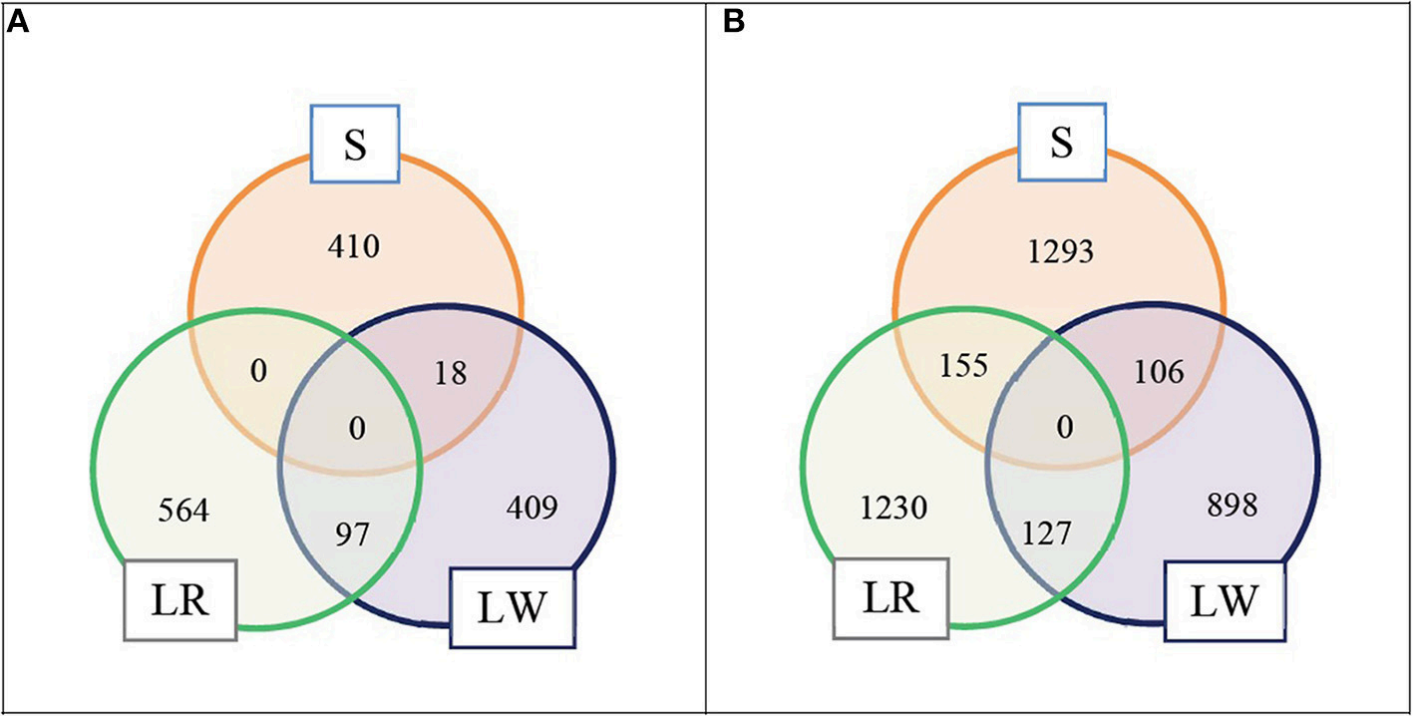
Numbers of selected SNP by breed of origin and the overlap between them. **(A)** For scenario 5% and **(B)** for scenario 10%.

We selected the top LD blocks that explained together either 5 or 10% of the total crossbred genetic variance for ADG in each breed of origin using the BOA model that treats all SNP in the same way. For the 5% scenario, for breed S origin there were 18 LD blocks which included in total 428 SNP; for breed LR origin there were 41 LD blocks which included in total 661 SNP, and for breed LW origin there were 26 LD blocks which included in total 524 SNP. These three groups of selected LD blocks per breed of origin were merged in one group, and after excluding duplicated SNP, resulted in 1,498 SNP classified as selected SNP. These selected SNP represent 3.6% of the whole SNP panel. The numbers of selected SNP by breed of origin and the overlap between them are illustrated in Figure 1A. For the 10% scenario, for breed S origin, there were 66 LD blocks which included in total 1,554 SNP; for breed LR origin, there were 109 LD blocks which included in total 1,512 SNP, and for breed LW origin, there were 73 LD blocks which included in total 1,131 SNP. These three groups of selected LD blocks per breed of origin were merged in one group, and after excluding the duplicated SNP, resulted in 3,809 SNP classified as selected SNP. These selected SNP represent 9.2% of the whole SNP panel. The numbers of selected SNP by breed of origin and the overlap between them are illustrated in Figure 1B.

### Variance Components, Heritabilities, and Genetic Correlations

Estimated variance components for ADG using the G, BOA, SEL-BOA 5%, and SEL-BOA 10% models are in Table 2. The standard errors of the estimated variance components in Table 2 are provided in Table S1. In the SEL-BOA 5% model, the selected SNP explained 39, 43, and 40% of the total crossbred genetic variance for S, LR, and LW, respectively. And for the SEL-BOA 10% model, the selected SNP explained 77, 75, and 79% of the total crossbred genetic variance for S, LR, and LW, respectively.

**Table 2:** Additive genetic variance ($\sigma_{a}^{2}$), litter variance ($\sigma_{u}^{2}$), residual variance ($\sigma_{e}^{2}$), and heritabilities for each breed for purebred (PB) and crossbred (CB) performance, and genetic correlation between PB and CB performance (***r***_***PC***_), estimated using G^a^, BOA^b^, and SEL-BOA^c^ models.

**Model**	**Breed**	$\sigma_{{\text{a}}_{\text{P}\text{B}}}^{2}$^*^		**$\sigma_{{\text{u}}_{\text{P}\text{B}}}^{2}$**	**$\sigma_{{\text{e}}_{\text{P}\text{B}}}^{2}$**	${\text{h}}_{\text{P}\text{B}}^{2}$^*^		$\sigma_{{\text{a}}_{\text{C}\text{B}}}^{2}$^*^		**$\sigma_{{\text{u}}_{\text{C}\text{B}}}^{2}$**	**$\sigma_{{\text{e}}_{\text{C}\text{B}}}^{2}$**	${\text{h}}_{\text{C}\text{B}}^{2}$^*^		**r**_pc_^*^	
G	S	2,686		3,205	9,271	0.18		2,284		1,404	4,168	0.29		0.66	
	LR	2,005		2,501	4,085	0.23								0.44	
	LW	2,320		2,278	5,664	0.23								0.49	
BOA	S	2,212		3,204	9,282	0.15		2,22		1,304	4,118	0.30^+^		0.73	
	LR	1,912		2,503	4,076	0.23		1,806						0.56	
	LW	2,135		2,276	5,669	0.21		2,883						0.62	
**Model**	**Breed**	$\sigma_{{\text{a}}_{\text{P}\text{B}}}^{2}$^*^		$\sigma_{{\text{u}}_{\text{P}\text{B}}}^{2}$	$\sigma_{{\text{e}}_{\text{P}\text{B}}}^{2}$	${\text{h}}_{\text{P}\text{B}}^{2}$^*^		$\sigma_{{\text{a}}_{\text{C}\text{B}}}^{2}$^*^		$\sigma_{{\text{u}}_{\text{C}\text{B}}}^{2}$	$\sigma_{{\text{e}}_{\text{C}\text{B}}}^{2}$	${\text{h}}_{\text{C}\text{B}}^{2}$^*^		**r**_pc_^*^	
		**Non-sel**	**Sel**			**Non-sel**	**Sel**	**Non-sel**	**Sel**			**Non-sel**	**Sel**	**Non-sel**	**Sel**
SEL-BOA 5%	S	2,064	410	3,230	9,310	0.14	0.03	1,357	866	1,363	4,154	0.17	0.12^+^	0.73	0.00
	LR	1,301	583	2,529	4,134	0.15	0.07		1,041					0.08	1.10
	LW	1,904	325	2,306	5,676	0.19	0.03		920					0.51	0.75
SEL-BOA 10%	S	1,596	798	3,200	9,349	0.11	0.05	543	1,777	1,370	4,088	0.07	0.23^+^	0.59	0.85
	LR	897	1,003	2,513	4,142	0.10	0.12		1,631					−0.25	1.14
	LW	1,648	592	2,307	5,674	0.16	0.06		2,039					0.61	0.81

*S, Synthetic boar, LR, Landrace (LR), LW, Large White (LW)*.

a *G model, model for across-breed effects for all SNP*.

b *BOA model, model for breed-specific effects for all SNP*.

c *SEL-BOA model, model with breed-specific effects for SNP strongly associated with crossbred performance and across-breed effects for all other SNP. SEL-BOA (5%) and SEL-BOA (10%) considering top 5% or top 10% of the SNP associated with crossbred performance as strongly associated with crossbred performance, respectively*.

* *SEL-BOA model has two estimates for $\sigma_{{\text{a}}_{\text{P}\text{B}}}^{\text{2}}$, $\sigma_{{\text{a}}_{\text{P}\text{B}}}^{\text{2}}$, and **r**_**pc**_, one for the across-breed component (Non-sel) and the other for breed-specific component (Sel)*.

+ *(0.5$\sigma_{a_{S}}^{\text{2}}$ +0.25$\sigma_{a_{LR}}^{\text{2}}$ + 0.25$\sigma_{a_{LW}}^{\text{2}}$)/(0.5$\sigma_{a_{S}}^{\text{2}}$ +0.25$\sigma_{a_{LR}}^{\text{2}}$ + 0.25$\sigma_{a_{LW}}^{\text{2}}$ + $\sigma_{u_{CB}}^{\text{2}}+$$\sigma_{e_{CB}}^{\text{2}}$)*.

Comparing purebred variance components across models, additive genetic variances were larger when estimated with the G model and smaller when estimated with the BOA model, while with the SEL-BOA models they were in between, but in general, estimates were similar across models. Likewise, heritability estimates were similar across models, around 0.17, 0.23, and 0.22 for S, LR, and LW, respectively. For the SEL-BOA, in this comparison the considered additive variance was obtained as the sum of the variance explained by the selected and non-selected SNP.

Comparing crossbred variance components across models, additive genetic variances were very similar across G (2284), BOA (2285), and SEL-BOA 5% (2,280) models, while the SEL-BOA 10% model had a slightly larger additive variance (2,349). For the BOA and SEL-BOA models, in this comparison the considered additive variance was obtained as the weighted sum of the variance explained by the selected and non-selected SNP, using weights of 0.50 for the paternal breed, and 0.25 for each of the maternal breeds. Crossbred heritabilities were similar across models (0.29–0.30).

Comparing crossbred genetic variance components by breed of origin, we observed similar estimates independent of the model used for breed S origin, however, for breed LR and LW origin, the estimates differed largely according to the model. The genetic correlations between performance of purebred and crossbred pigs (r_pc_) estimated with the G model did not differ largely from the r_pc_ estimated with the BOA model for breed S origin. A larger difference was observed for the maternal breeds LR and LW, however, differences in r_pc_ between the models were within the range of the standard errors. With the SEL-BOA models, the r_pc_ for the selected SNP was larger than for the non-selected SNP, except for breed S origin when calculated with the SEL-BOA 5% where the r_pc_ for the selected SNP was zero. For breed LR origin, the estimate of the r_pc_ for the selected SNP was larger than unity with both, the SEL-BOA 5% and the SEL-BOA 10%, for further analysis we fixed the value to 0.99. For breed LR origin, the r_pc_ for the non-selected SNP calculated with the SEL-BOA 10%, had a value lower than zero and large SE (±0.31). Although this value is theoretically possible, for further analysis we fixed it to zero. For LR breed, the r_pc_ for non-selected SNP and selected SNP estimated with the SEL-BOA 5% and SEL-BOA 10% were very different compared to the r_pc_ estimated with the G or BOA model. For S and LW breeds, there was not a big discrepancy for the r_pc_ estimates across models, except for the r_pc_ of zero estimated for the selected SNP with SEL-BOA 5% model. In general, SE of r_pc_ increased as the models gained complexity (see Table S1). When SNP were split between selected SNP and non-selected SNP, as the number of SNP increased in one of the groups the SE decreased, or the other way around, as the number of SNP decreased in one of the groups the SE increased. Estimates for the r_pc_ of the LR line, being lower than 0 for the non-selected and >1 for the selected SNPs, suggest that the size of the dataset was limiting the accuracy of the estimated variance components. Crossbred heritability estimates for the SEL-BOA 5% were higher for the non-selected SNP (0.17) than for the selected SNP (0.12). Conversely, crossbred heritability estimates for SEL-BOA 10% were lower for the non-selected SNP (0.07) than for the selected SNP (0.23).

### Predictive Ability for Breeding Values

Accuracies of the four models for GEBV of purebred pigs for crossbred performance for ADG are in Table 3. DRP for purebred pigs were used as purebred phenotypes for crossbred performance in the validation. On average, DRP calculated for LR animals were more reliable (i.e., they had a higher *w*) than the DRP calculated for S and LW animals. This is considered when computing the accuracies as we used *w* to weight the correlations. In general the differences in accuracies between the models were small, but there was a tendency that the SEL-BOA 10% model performed better than the other models, at least for the paternal breed S and maternal breed LR. For the maternal breed LW, similar accuracies were obtained with the four models.

**Table 3:** Accuracies^*^ of G^a^, BOA^b^, and SEL-BOA^c^ models calculated for estimating breeding values of purebred pigs for crossbred performance for each of the 4-folds of cross-validation and average weighting factor (*w*) of the calculated DRP per validation fold.

**Folds**	***w***	**G**	**BOA**	**SEL-BOA 5%**	**SEL-BOA 10%**
**S**					
1	0.15	0.132	0.118	0.116	0.129
2	0.18	0.024	0.030	0.026	0.038
3	0.23	0.119	0.119	0.112	0.145
4	0.09	0.092	0.092	0.092	0.100
*Mean*		*0.092*	*0.090*	*0.086*	*0.103*
**LR**					
1	0.26	0.140	0.177	0.110	0.152
2	0.25	0.166	0.167	0.153	0.204
3	0.21	0.111	0.105	0.183	0.175
4	0.24	0.172	0.163	0.173	0.202
*Mean*		*0.147*	*0.153*	*0.155*	*0.183*
**LW**					
1	0.20	0.150	0.163	0.158	0.149
2	0.16	0.138	0.133	0.133	0.135
3	0.11	0.123	0.135	0.118	0.114
4	0.21	0.149	0.160	0.154	0.155
*Mean*		*0.140*	*0.148*	*0.141*	*0.134*

* *Accuracies measured as weighted correlation between DRP and EBVs*.

*S, Synthetic boar; LR, Landrace (LR); LW, Large White (LW)*.

a *G model, model for across-breed effects for all SNP*.

b *BOA model, model for breed-specific effects for all SNP*.

c *SEL-BOA model, model with breed-specific effects for SNP strongly associated with crossbred performance and across-breed effects for all other SNP. SEL-BOA 5% and SEL-BOA 10% considered top 5% or top 10% of the SNP associated with crossbred performance as strongly associated with crossbred performance, respectively*.

*Approximate standard errors SE, computed as $\frac{1-r^{\text{2}}}{\sqrt{N}}$, were equal to 0.035–0.036 for the mean accuracies across the folds, for all methods*.

## Discussion

The objective of this study was to develop a model that accounts for breed-specific allele effects only for SNP strongly associated with crossbred performance, and for the rest of the SNP assumes that effects are the same across breeds.

To construct the relationship matrices for the SEL-BOA model, we selected SNP that explained together at the most either 5 or 10% of the total genetic variance in each breed of origin using the BOA model. Dominance was not considered in the BOA model, neither in the G model nor the SEL-BOA model. However, breed-specific allele substitution effects were estimated based on commercial crossbred performance allowing the effects to be estimated under the genetic background in which they are expressed. Thus, if dominance effects are present, estimated breed-specific allele substitution effects incorporate the heritable component of dominance, even if dominance effects are not modeled explicitly.

Moreover, when estimating SNP allele effects with the BOA model, the effect of alleles based on breed of origin was confounded with parental origin. For ADG, there is evidence of QTL that exhibit parental-origin-specific effects (de Koning et al., 2001) and it has been shown that genomic imprinting significantly contributes to the genetic variance (Neugebauer et al., 2010). The evidence for QTL exhibiting parental-origin-specific effects, suggests that having one instead of both reciprocal female crosses would likely affect the list of selected SNP. For the F1 females, in practice both reciprocal crosses are used, and therefore also both were included in our data. Effectively, this means that SNP were likely selected even if their effect was limited to the allele inherited from a parent of a specific sex, unless the absence of an effect when inherited from the other parent diluted the association to the trait too much.

In the SEL-BOA 10% model the selected SNP actually explained 77, 75, and 79% of the total additive genetic variance for S, LR, and LW, respectively. This shows that the SEL-BOA model was really able to attribute much more genetic variance to the selected SNP than the original BOA model, where all SNP were treated similarly in the model. These high percentages of explained variance left little crossbred additive genetic variance to be explained by the non-selected SNP, so we did not pursue any scenarios that selected even more SNP.

Across the models, the heritability for crossbred performance was very similar. However, the models using breed of origin of alleles (BOA, SEL-BOA 5%, and SEL-BOA 10%) showed that estimates of crossbred additive genetic variance differed between the three breeds. This suggests that the G model, on average, obtains the correct heritability, even if the contribution to the crossbred variance of the different breeds varies. In theory, the crossbred additive variance components estimated with the BOA model comprises the variance observed in crossbred pigs due only to the alleles coming from the analyzed breed. This implies that the breed-specific r_pc_ values estimated with the BOA model are effectively correlations of effects on purebred and crossbred performance of alleles originating from the same breed, while the G model estimates r_pc_ values considering effects of alleles originating from all breeds involved in the crossbred. Therefore, r_pc_ are expected to be higher when calculated with the BOA model rather than the G model, and this is also what we observed in our estimates. For breed S, estimated crossbred genetic variance and r_pc_ were very similar between the G and BOA model, and no benefit for calculating GEBV of S purebred animals for crossbred performance was observed using the BOA model. However, a benefit was observed for breeds LR and LW that showed larger differences in their estimates of crossbred genetic variance and r_pc_ between the G and BOA model. Similar results were found by Sevillano et al. (2017) who used similar but smaller data sets.

With the SEL-BOA models, the r_pc_ for non-selected SNP are calculated as in the G model, while the r_pc_ for selected SNP are calculated by breed of origin as in the BOA model, therefore, as explained in the previous paragraph, we could also expect that the r_pc_ for selected SNP is higher than the r_pc_ for non-selected SNP. However, in the SEL-BOA models, the selected SNP are chosen to be SNP strongly associated with crossbred performance, but if those SNP have a different estimated effect for purebred performance the r_pc_ for selected SNP may actually be smaller. Overall, we observed a tendency of r_pc_ being greater for selected than non-selected SNP.

The SEL-BOA models have potentially two advantages, arising from having separate variance components for the selected and non-selected SNP. Firstly, SEL-BOA models are able to assign more variance to SNP with a strong association to the trait than the G and BOA models, and less to the non-selected SNP. Secondly, they can differentiate the r_pc_ values for the two categories of SNP. Differences in variance estimates alone are not sufficient to cause a difference in accuracy, the benefit of the SEL-BOA model comes when r_pc_ estimates are also different. For instance, for breeds LR and LW, the crossbred genetic variance estimated for non-selected and selected SNP estimated with the SEL-BOA 5% and SEL-BOA 10% were very different compared to the crossbred genetic variance estimated with the G or BOA model. However, for LW, there were not large differences across the estimates of r_pc_, subsequently, no benefit of the SEL-BOA models were observed. Conversely, for LR, the r_pc_ for non-selected and selected SNP estimated with the SEL-BOA 5% and SEL-BOA 10% were very different compared to the r_pc_ estimated with the G or BOA model. The estimated r_pc_ for the selected SNP was >1, and we assumed a value of 0.99 in the subsequent analyses, meaning that their estimated effects are similar for purebred and crossbred performance. On the other hand, the r_pc_ for the non-selected SNP was below zero, and we assumed a value of zero in the subsequent analyses. This means that their estimated effects for purebred and crossbred performance are totally different, and using crossbred information is needed for estimating effects for crossbred performance as it cannot be derived from purebred information. As a result, SEL-BOA models were more accurate for calculating GEBV of LR purebred animals for crossbred performance than the BOA or G models.

For breed S, similar to breed LW, accuracies for calculating GEBV of S purebred animals for crossbred performance were similar between the SEL-BOA models and the other models. For these breeds, there was not a big discrepancy for the r_pc_ estimates, except for the r_pc_ estimated for the selected SNP with SEL-BOA 5% model. In this case, however, the impact might not be so high because the selected SNP only represented 39% of the crossbred genetic variance, therefore the main genetic variance was due to the non-selected SNP that had an r_pc_ that was close to the estimates of the BOA and G models. In general, the differences were small, which may in part be because the SEL-BOA models actually had lower power than the G model because of the larger number of effects fitted. In general this is a problem that is faced by all models using the concept of breed of origin of alleles (Ibánez-Escriche et al., 2009, Vandenplas et al., 2017).

Although with the SEL-BOA 5% the selected SNP explained 39, 43, and 40% of the total crossbred genetic variance for S, LR, and LW, respectively, this model performed similar to the G model for S and LW. For LR, allowing the 1,498 selected SNP to have a different effect rather than effects estimated combining the other breeds S and LW, improved accuracy. An important question is why LR did seem to benefit from using the SEL-BOA model, while S and LW did not. It is good to note that the S breed was created as a combination of Large White and Pietrain, which suggests that the S and LW breed, a Large White based dam line, are somehow more related than the other breed pairs. On the other hand LR is a Landrace based dam line and LR pigs have undergone a different selection pressure that may have shaped their genomic architecture differently, possibly resulting partly in different haplotypes, and different haplotypes frequencies for the haplotypes that are in common with the other breeds (Egbert Knol, personal communication). In a previous study, Sevillano et al. (2018) observed that the explained genetic variance of haplotypes associated to the MC4R gene, which has a missense mutation with a known effect on ADG (Kim et al., 2000), was considerably lower for the LR and also this breed showed the lowest allele frequency of the mutation compared to breed S and LW. This seems to confirm that the LR breed indeed is quite different from the S and LW breeds. Similar to the MC4R, other regions coming from the LR breed might also show different genetic variance compared to S and LW, providing a possible explanation why this breed shows some benefit when some SNP are allowed to be estimated separately by breed of origin in the SEL-BOA 5%. With the SEL-BOA 10%, the benefit for LR breed is even larger. With the SEL-BOA 10% model the benefit of the BOA model is obtained while reducing possible disadvantages due to calculating three times as many effects, because breed of origin specific effects are estimated for fewer SNP.

The implementation of the SEL-BOA model for routine genetic evaluations faces the challenge of multi-trait analysis. In a multi-trait analysis, many traits are included in the model, and they are analyzed with the same relationship matrix or with the same partial relationship matrices. Therefore, in the SEL-BOA model, the partial relationship matrices with SNP selected to have breed-specific allele effects will not be trait-specific, but this can be overcome by defining one group of selected SNP for all traits, which includes all SNP associated with at least one of the traits. This means that per trait more SNP, including some SNP not associated with the target trait, will be used to estimate breed-specific variance components and effects. As more SNP are allowed to have breed-specific allele effects, the benefit of making a distinction between non-selected SNP and selected SNP will be diluted, and the accuracy of the SEL-BOA model will decay. To minimize this disadvantage, SEL-BOA depends on the advancement of association studies to select SNP only highly associated with one or more traits of interest.

## Conclusions

The BOA model was more accurate for calculating GEBV of purebred animals for crossbred performance than the G model when estimated crossbred genetic variances and r_pc_ differed largely between the G model and the BOA model. Superiority of the SEL-BOA model compared to the BOA model was only observed for the SEL-BOA model 10% when r_pc_ for the non-selected SNP and selected SNP differed strongly from the r_pc_ estimated by the BOA model.

## Author Contributions

CS has designed the study, prepared the data, conducted the analyses, prepared figures and tables, and wrote the first draft of the manuscript. HB participated in the discussion of analysis issues. MC participated in the design of the study and coordination, was involved in the construction and evaluation of the model, and the discussion of analysis issues. All authors contributed to manuscript revision, read, and approved the submitted version.

### Conflict of Interest Statement

The authors declare that the research was conducted in the absence of any commercial or financial relationships that could be construed as a potential conflict of interest.
