# Supplementary material for: Genomic Evaluation for a Crossbreeding System Implementing Breed-of-Origin for Targeted Markers
Source: Front Genet. 2019 May 3;10:418. doi: 10.3389/fgene.2019.00418 (PMC6510010; doi:10.3389/fgene.2019.00418)
Supplement: Supplementary file 1 [file Table_1.DOCX]

Supplementary Material

Genomic evaluation for a crossbreeding system implementing breed-of-origin for targeted markers

Claudia A Sevillano*, Henk Bovenhuis, Mario PL Calus

*** Correspondence:** Claudia A. Sevillano: claudia.sevillanodelaguila@wur.nl

# Supplementary Data

## Standard errors of additive genetic variance ($\boldsymbol{\sigma}_{\boldsymbol{a}}^{\boldsymbol{2}}$ ), litter variance ($\boldsymbol{\sigma}_{\boldsymbol{u}}^{\boldsymbol{2}}$), residual variance ($\boldsymbol{\sigma}_{\boldsymbol{e}}^{\boldsymbol{2}}$), and heritabilities for each breed for purebred (PB) and crossbred (CB) performance, and genetic correlation between PB and CB performance ($\boldsymbol{r}_{\boldsymbol{PC}}$), estimated using the G^a^, BOA^b^, and SEL-BOA^c^ models.

| **Model** | **Breed** | $\boldsymbol{\sigma}_{\mathbf{a}_{\mathbf{PB}}}^{\mathbf{2}}$***** | | $\boldsymbol{\sigma}_{\mathbf{u}_{\mathbf{PB}}}^{\mathbf{2}}$ | $\boldsymbol{\sigma}_{\mathbf{e}_{\mathbf{PB}}}^{\mathbf{2}}$ | $\boldsymbol{h}_{\mathbf{PB}}^{\boldsymbol{2}}$***** | | $\boldsymbol{\sigma}_{\mathbf{a}_{\mathbf{CB}}}^{\mathbf{2}}$***** | | $\boldsymbol{\sigma}_{\mathbf{u}_{\mathbf{CB}}}^{\mathbf{2}}$ | $\boldsymbol{\sigma}_{\mathbf{e}_{\mathbf{CB}}}^{\mathbf{2}}$ | $\boldsymbol{h}_{\mathbf{CB}}^{\boldsymbol{2}}$***** | | $\mathbf{r}_{\mathbf{pc}}$***** | |
| --- | --- | --- | --- | --- | --- | --- | --- | --- | --- | --- | --- | --- | --- | --- | --- |
| G | S | 273.7 | | 216.2 | 199.1 | 0.016 | | 284.2 | | 199.3 | 167.8 | 0.031 | | 0.081 | |
|  | LR | 253.9 | | 215.9 | 178.9 | 0.026 | |  |  |  |  |  |  | 0.118 | |
|  | LW | 159.1 | | 112.8 | 94.3 | 0.013 | |  |  |  |  |  |  | 0.081 | |
| BOA | S | 226.9 | | 216.2 | 199.1 | 0.014 | | 375.8 | | 200.5 | 183.3 | 0.30^+^ | | 0.086 | |
|  | LR | 241.3 | | 216.2 | 178.7 | 0.025 | | 646.0 | |  |  |  |  | 0.177 | |
|  | LW | 146.5 | | 112.7 | 94.3 | 0.012 | | 687.5 | |  |  |  |  | 0.103 | |
| **Model** | **Breed** | $\boldsymbol{\sigma}_{\mathbf{a}_{\mathbf{PB}}}^{\mathbf{2}}$***** | | $\boldsymbol{\sigma}_{\mathbf{u}_{\mathbf{PB}}}^{\mathbf{2}}$ | $\boldsymbol{\sigma}_{\mathbf{e}_{\mathbf{PB}}}^{\mathbf{2}}$ | $\boldsymbol{h}_{\mathbf{PB}}^{\boldsymbol{2}}$***** | | $\boldsymbol{\sigma}_{\mathbf{a}_{\mathbf{CB}}}^{\mathbf{2}}$***** | | $\boldsymbol{\sigma}_{\mathbf{u}_{\mathbf{CB}}}^{\mathbf{2}}$ | $\boldsymbol{\sigma}_{\mathbf{e}_{\mathbf{CB}}}^{\mathbf{2}}$ | $\boldsymbol{h}_{\mathbf{CB}}^{\boldsymbol{2}}$***** | | $\mathbf{r}_{\mathbf{pc}}$***** | |
|  |  | **Non-sel** | **Sel** |  |  | **Non-sel** | **Sel** | **Non-sel** | **Sel** |  |  | **Non-sel** | **Sel** | **Non-sel** | **Sel** |
| m-BOA 5% | S | 259.3 | 122.5 | 216.6 | 199.3 | 0.016 | 0.008 | 253.5 | 253.1 | 194.1 | 166.6 | 0.03 | 0.03 | 0.114 | 0.002 |
|  | LR | 233.2 | 146.9 | 215.7 | 177.6 | 0.026 | 0.017 |  | 359.5 |  |  |  |  | 0.177 | 0.126 |
|  | LW | 149.3 | 73.8 | 113.3 | 94.2 | 0.013 | 0.007 |  | 350.0 |  |  |  |  | 0.109 | 0.161 |
| m-BOA 10% | S | 251.8 | 167.6 | 215.5 | 199.6 | 0.016 | 0.011 | 217.9 | 340.6 | 190.9 | 163.3 | 0.020 | 0.035 | 0.208 | 0.101 |
|  | LR | 226.6 | 195.4 | 214.2 | 176.3 | 0.026 | 0.022 |  | 453.6 |  |  |  |  | 0.305 | 0.116 |
|  | LW | 146.1 | 98.8 | 113.3 | 94.1 | 0.013 | 0.009 |  | 496.6 |  |  |  |  | 0.188 | 0.111 |

S = Synthetic boar, LR = Landrace (LR), LW = Large White (LW).

^a^$G$ model, model for across-breed effects for all SNPs

^b^BOA model, model for breed-specific effects for all SNPs.

^c^SEL-BOA model, model with breed-specific effects for SNPs strongly associated with crossbred performance and across-breed effects for all other SNPs. SEL-BOA (5%) and SEL-BOA (10%) considering top 5% or top 10% of the SNPs associated with crossbred performance as strongly associated with crossbred performance, respectively.
